# The treatment pattern and adherence to direct oral anticoagulants in patients with atrial fibrillation aged over 65

**DOI:** 10.1371/journal.pone.0214666

**Published:** 2019-04-01

**Authors:** Sola Han, Hwa Seop Jeong, Hyungtae Kim, Hae Sun Suh

**Affiliations:** College of Pharmacy, Pusan National University, Busan, Korea; Maastricht University Medical Center, NETHERLANDS

## Abstract

**Objective:**

In this study, we aimed to assess the utilization pattern (potentially inappropriate dosing and concomitant use of contraindicated drugs) and adherence to direct oral anticoagulants (DOACs), including apixaban, dabigatran, and rivaroxaban, in patients with atrial fibrillation (AF) unsuitable for warfarin.

**Methods:**

We used nationally representative data, namely Health Insurance Review and Assessment Service-Aged Patient Sample 2014, that included medical and pharmacy claims of approximately 1 million patients aged 65 or older. We included patients who had at least one diagnosis of AF and at least one prescription of DOAC between January 1 and December 31, 2014. In 2014, DOACs were reimbursed only to patients with AF unsuitable for warfarin. Appropriate dosing and contraindicated drugs were determined according to the Summary of Product Characteristics for each DOAC. Multivariate logistic regression was performed to examine the factors contributing to the concomitant use of contraindicated drugs. To assess adherence, we calculated the medication possession ratio (MPR).

**Results:**

The percentage of inappropriate dosing was 11.8% among 1,234 patients with AF; it was the highest in rivaroxaban users (16.8%). Contraindicated drugs were prescribed to 236 patients (19.1%). Clinics, smaller healthcare institutions, and outpatient visits were significantly related to contraindicated drug use. The mean MPRs were 0.95, 0.93, and 0.91 for apixaban, dabigatran, and rivaroxaban, respectively (*P* = 0.075).

**Conclusions:**

Careful monitoring is warranted in patients with AF aged over 65 who were unsuitable for warfarin to reduce the incidence of inappropriate dosing and concomitant use of contraindicated drugs.

## Introduction

Atrial fibrillation (AF) is one of the risk factors of stroke, where 15–20% of stroke events are related to AF [1, 2]. Especially, older patients with AF constitute almost half of all patients with AF and are known to have increased risk of stroke and bleeding [3]. As a previous study reported, doses of oral anticoagulants (OACs), concomitant drugs, comorbidities, and adherence largely affect the anticoagulant effect of OACs in older patients with AF [3].

Warfarin has been widely used for prevention of stroke in patients with AF. Based on the results of several clinical trials, direct oral anticoagulants (DOACs) were also approved for the same purpose [4–7]. Compared to warfarin, DOACs have fewer drug interactions and lower risk of intracranial hemorrhage. In addition, it does not require regular blood tests [8].

Until July 2015, DOACs were reimbursed by the National Health Insurance of Korea only to patients with AF who were at high risk of development of stroke (i.e., CHA_2_DS_2_-VASc score of two or more) and unsuitable for warfarin. Although DOACs have been used in Korea for almost 6 years, little is known about the real-world use, particularly the drug utilization patterns and medication adherence, in Korea, which is important to develop strategies for optimal stroke prevention.

Therefore, in this study, we aimed to assess the drug utilization patterns and medication adherence to DOACs among patients with AF aged over 65 who were unsuitable for warfarin in a real-world setting. Drug utilization patterns included potentially inappropriate dosing, switching, and concomitant use of contraindicated drugs.

## Methods

### Data source

We used nationally representative data, namely Health Insurance Review and Assessment Service-Aged Patient Sample (HIRA-APS) 2014 in this study. This data included medical and pharmacy claims of approximately 1 million patients aged 65 or older, who represented 20% of all Korean patients aged over 65 [9]. All patient data in HIRA-APS were fully anonymized to ensure privacy. Data included the following variables: age, sex, insurance information, diagnosis codes, prescriptions and medical institution information. Diagnoses were coded according to the *International Classification of Diseases*, *Tenth Revision* (ICD-10) [9]. The Pusan National University Institutional Review Board found that this study was exempt from ethical review (PNU IRB/2016_103_HR).

### Study subjects

We included patients who had at least one diagnosis of AF (ICD-10 code, *I48*.*0*) and at least one prescription of DOACs (apixaban, dabigatran, or rivaroxaban) during the study period between January 1, 2014 and December 31, 2014. For reference, in 2014 in Korea, 2006–2008 version of ICD-10 code was used. In this version of ICD-10, I48 included only I48.0 (atrial fibrillation) and I48.1 (atrial flutter), but not as many subcodes as recent version of ICD-10 does.

The type of the first prescribed DOAC was defined as the index DOAC. The index date was defined as the date of the first DOAC prescription with AF diagnosis code. Medication codes used in this study are listed in S1 Table. Included patients were patients with AF unsuitable for warfarin due to poor international normalized ratio (INR) control, hypersensitivity, or contraindication to warfarin, according to the Korean reimbursement criteria for DOACs in 2014. We excluded patients who had two or more DOACs at the index date. We followed up the patients from the index date until one of the following three occurrences, whichever comes first: (1) the first occurrence of switching, (2) the last date of DOAC prescription plus days of supply for the last prescription, or (3) the study end date (December 31, 2014). We identified the age, sex, insurance type, level of institution, bed size and region of institution, and type of visit (inpatient or outpatient) at the index date as the baseline characteristics of the study subjects. We also calculated the CHA_2_DS_2_-VASc score, AnTicoagulation and Risk Factors in Atrial Fibrillation score (ATRIA score), and Charlson Comorbidity Index (CCI) by using ICD-10 codes at the index date (S2, S3 and S4 Tables).

### Drug utilization patterns

We assessed potentially inappropriate dosing, switching, and concomitant use of contraindicated drugs. To assess potentially inappropriate dosing, we used the daily doses regardless of the frequency of dosing per day. Then, we divided the patients into two groups to define them as having potentially inappropriate dosing, (1) patients with DOAC dosage higher than standard dose; and (2) patients with DOAC dosage lower than recommended low dose. The standard dose was defined as the maintenance dose recommended for patients with AF and the recommended low dose was defined as the reduced dose recommended for patients with AF whose health status (i.e., weight, age, and kidney function) was not appropriate for receiving standard dose (S5 Table) [10–13]. We also identified patients with total hip or knee replacement, deep venous thrombosis or pulmonary embolism by using Korean procedure codes for hip and knee replacement surgery and ICD-10 codes of deep venous thrombosis and pulmonary embolism (S6 Table), because their anticoagulation strategies were different from those in patients with AF. Thus, we ignored the following doses to make them not to be considered as any potentially inappropriate dosing: (1) doses for patients with hip and knee replacement surgery (until 5 and 2 weeks after hip and knee replacement surgery, respectively); (2) doses for patients with acute deep venous thrombosis or pulmonary embolism (until 3 weeks after its occurrence). In addition, if patients received doses other than the recommended doses for ≤ 7 days, these doses were not considered as a potentially inappropriate dosing, because we assumed that this was a dose adjustment period.

Switching was defined as initiation of another DOAC and its use for ≥ 8 days to minimize the possibility that a short-term overlapping prescription period for switching would be considered as a concomitant use. Contraindicated drugs were defined according to the Summary of Product Characteristics (SmPC) for each DOAC. The medication codes of contraindicated drugs used in this analysis are found in S7 Table. We excluded unfractionated heparin (UFH) from the analysis because it could be used in combination with DOACs when patients should maintain a patent central venous or arterial catheter. We also excluded systemic ketoconazole (P-glycoprotein [P-gp] inhibitor) from the analysis because it was withdrawn from the Korean market in 2013. For P-gp inducers, they have a substantial impact on the plasma concentrations of all DOACs according to the SmPC, they were considered as a contraindication only to dabigatran according to the package insert in Korea. The concomitant use of contraindicated drugs was defined as the filling of a prescription of contraindicated drug during the period in which DOAC was also prescribed. However, the concomitant use of other OACs prescribed for ≤ 7 days was not considered as a concomitant use of contraindicated drugs.

### Medication adherence

Medication adherence to DOACs was assessed by calculating the medication possession ratio (MPR). The MPR was calculated by dividing the number of days of medication supplied within the refill interval by the number of days in the refill interval [14]. Based on the taxonomy of adherence to medication (i.e., initiation, implementation, and discontinuation) [15], we evaluated adherence of patients with at least two DOAC prescriptions (i.e., the implementation phase of adherence). If the calculated MPR was ≥ 0.8, it was considered an adherent case. If the calculated MPR was ˃ 1, it was considered 1. If switching occurred, we only included the prescriptions of index DOACs.

### Statistical analysis

The baseline characteristics of the study subjects were expressed as the means ± standard deviations for continuous variables and frequency with percentage for categorical variables. The differences in the baseline characteristics and results (drug utilization patterns and adherence) were analyzed using analysis of variance (ANOVA) followed by Bonferroni *post-hoc* test for continuous variables and χ^2^ or Fisher’s exact test for categorical variables. Logistic regression analysis was used to assess the factors associated with the concomitant use of contraindicated drugs. To check the performance of the logistic model, we examined the *c*-statistic. In all statistical analyses, a *P*-value of < 0.05 was considered statistically significant.

## Results

### Study population

**Fig 1:**
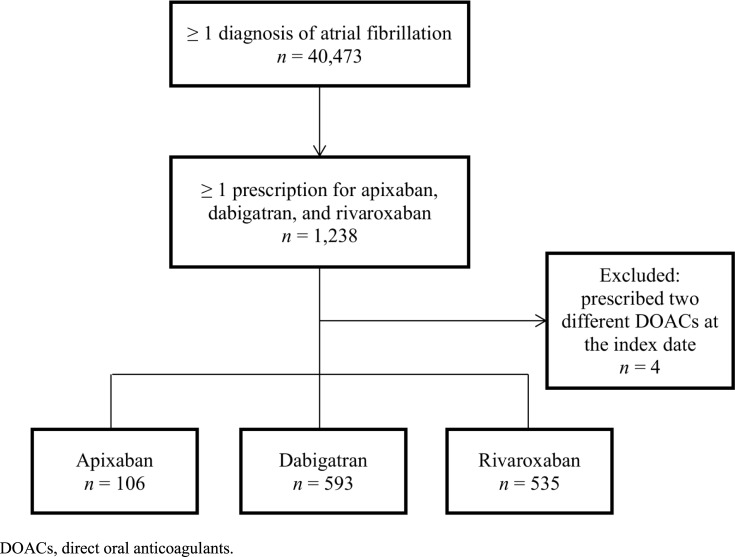
Flow chart of patient selection.

We identified 40,473 patients with AF, of whom 1,234 patients fulfilled our inclusion criteria for the drug utilization pattern analysis. Fig 1 shows the process of patient selection. The baseline characteristics of the patients stratified according to index DOACs are shown in Table 1. The majority of patients were treated with dabigatran (48.1%), followed by rivaroxaban (43.4%) and apixaban (8.6%). The overall mean age was 76.2 years. Dabigatran users (75.7 years) were significantly younger, compared to rivaroxaban users (76.9 years, *P* = 0.003). Approximately half of the patients had cerebrovascular disease in the three groups receiving different DOACs. Approximately 28.3–34.0% of the patients had congestive heart failure, and 20.9–30.2% were diagnosed with diabetes without complications. S1 Fig (CHA_2_DS_2_-VASc) and S2 Fig (ATRIA) show the distribution of patients receiving each DOAC. There was no significant difference in predicted stroke risk associated with different DOACs, whereas apixaban and rivaroxaban users had a significantly higher predicted bleeding risk, compared to dabigatran users (*P* = 0.005).

**Table 1:** Demographic characteristics of the study population.

	Total, *n* (%)	Apixaban, *n* (%)	Dabigatran, *n* (%)		Rivaroxaban, *n* (%)	*P* value
***Overall*, *n***	1234	106	593		535	
***Sex***						< 0.001*
Female	615 (49.8)	42 (39.6)	273 (46.0)		300 (56.1)	
Male	619 (50.2)	64 (60.4)	320 (54.0)		235 (43.9)	
***Age*, *mean ± SD***	76.2 ± 6.09	75.8 ± 5.98	75.7 ± 5.93		76.9 ± 6.23	0.003* ^a^
***Insurance type***						0.107
NHI	1108 (89.8)	95 (89.6)	533 (89.9)		480 (89.7)	
Medical Aid	104 (8.4)	11 (10.4)	44 (7.4)		49 (9.2)	
PVI	22 (1.8)	0 (0)	16 (2.7)		6 (1.1)	
***Level of institution***						< 0.001*
Clinic	49 (4.0)	4 (3.8)	28 (4.7)		17 (3.2)	
Long-term care hospital	10 (0.8)	0 (0)	8 (1.3)		2 (0.4)	
Hospital	105 (8.5)	0 (0)	30 (5.1)		75 (14.0)	
General hospital	629 (41.9)	33 (31.1)	278 (46.9)		205 (38.3)	
Specialized general hospital	556 (44.9)	69 (65.1)	249 (42.0)		236 (44.1)	
***Bed size***						< 0.001*
≤ 100	66 (5.3)	4 (3.8)	33 (5.6)		29 (5.4)	
101–300	168 (13.6)	2 (1.9)	56 (9.4)		110 (20.6)	
301–800	507 (41.1)	40 (37.7)	282 (47.6)		185 (34.6)	
801–1000	235 (19.0)	29 (27.4)	118 (19.9)		88 (16.4)	
>1,000	258 (20.9)	31 (29.2)	104 (17.5)		123 (23.0)	
***Region of institution***						0.001*
Seoul ∙ Gyeonggi	616 (49.9)	68 (64.2)	290 (48.9)		258 (48.2)	
Gangwon	45 (3.6)	4 (3.8)	26 (4.4)		15 (2.8)	
Chungcheong	118 (9.6)	8 (7.5)	63 (10.6)		47 (8.8)	
Gyeongsang	237 (19.2)	18 (17.0)	96 (16.2)		123 (23.0)	
Jeolla	205 (16.6)	8 (7.5)	115 (19.4)		82 (15.3)	
Jeju	13 (1.1)	0 (0)	3 (0.5)		10 (1.9)	
***Type of hospital visit***						< 0.001*
Inpatient	257 (20.8)	14 (13.2)	80 (13.5)		163 (30.5)	
Outpatient	977 (79.2)	92 (86.8)	513 (86.5)		372 (69.5)	
***Comorbidities***						
Cerebrovascular disease	679 (55.0)	58 (54.7)	382 (64.4)		239 (44.7)	< 0.001*
Congestive heart failure	356 (28.8)	36 (34.0)	168 (28.3)		152 (28.4)	0.478
Chronic pulmonary disease	141 (11.4)	6 (5.7)	55 (9.3)		80 (15.0)	0.02*
Dementia	174 (14.1)	11 (10.4)	86 (14.5)		77 (14.4)	0.514
Diabetes without chronic complication	275 (22.3)	32 (30.2)	131 (22.1)		112 (20.9)	0.111
Mild liver disease	89 (7.2)	2 (1.9)	40 (6.7)		47 (8.8)	0.036*
Myocardial infection	30 (2.4)	1 (0.9)	11 (1.9)		18 (3.4)	0.151
Peripheral vascular disease	61 (4.9)	7 (6.6)	21 (3.5)		33 (6.2)	0.090
Peptic ulcer disease	93 (7.5)	7 (6.6)	43 (7.3)		43 (8.0)	0.821
Rheumatologic disease	11 (0.9)	2 (1.9)	3 (0.5)		6 (1.1)	0.211^b^
Diabetes with chronic complication	66 (5.3)	9 (8.5)	31 (5.2)		26 (4.9)	0.311
Hemiplegia or paraplegia	56 (4.5)	3 (2.8)	36 (6.1)		17 (3.2)	0.045*
Any malignancy, including leukemia and lymphoma	55 (4.5)	4 (3.8)	17 (2.9)		34 (6.4)	0.017*
Renal disease	26 (2.1)	8 (7.5)	8 (1.3)		10 (1.9)	< 0.001*
Moderate or severe liver disease	2 (0.2)	0 (0)	1 (0.2)		1 (0.2)	1.000^b^
AIDS/HIV	2 (0.2)	0 (0)	0 (0)		2 (0.4)	0.352^b^
Metastatic solid tumor	6 (0.5)	2 (1.9)	0 (0)		4 (0.7)	0.014*
***Risk score*, *mean ± SD***						
CHA_2_DS_2_-VASc	4.51 ± 1.33	4.55 ± 1.35	4.51 ± 1.25		4.50 ± 1.42	0.956
ATRIA	2.21 ± 1.53	2.53 ± 1.69	2.08 ± 1.42		2.30 ± 1.59	0.005*^c^
CCI	1.91 ± 1.56	2.09 ± 2.15	1.90 ± 1.38		1.91 ± 1.61	0.483

SD, standard deviation; PVI, patriots & veterans insurance; CHA_2_DS_2_-VASc, score based on congestive heart failure, hypertension, age ≥ 75 years, diabetes mellitus, stroke, vascular disease, age 65–74 years, and sex category; ATRIA, AnTicoagulation and Risk Factors In Atrial Fibrillation; CCI, Charlson Comorbidity Index.

*Statistically significant, *P* < 0.05

^a^*P* = 0.002 for dabigatran *versus* rivaroxaban, *P* > 0.05 for apixaban *versus* dabigatran, and *P* > 0.05 for apixaban *versus* rivaroxaban based on Bonferroni *post-hoc* test

^b^Fisher’s exact test was used

^c^*P* = 0.017 for apixaban *versus* dabigatran, *P* = 0.049 for dabigatran *versus* rivaroxaban, and *P* > 0.05 for apixaban *versus* rivaroxaban based on Bonferroni *post-hoc* test.

### Drug utilization patterns

S3 Fig shows the number of AF patients who were prescribed DOACs monthly in 2014. Dabigatran was the most prescribed DOAC in Korea. There was a slight increase in the number of patients using apixaban and rivaroxaban over 2014.

The percentage of potentially inappropriate dosing and switching are shown in Table 2. The percentage of potentially inappropriate dosing was the highest in the rivaroxaban group (16.8%), followed by the dabigatran and apixaban groups (9.3 and 0.9%, respectively; *P* < 0.001). Among the two types of inappropriate dosing, the use of a dose lower than the recommended low dose was more frequent than the use of a dose higher than standard dose. The percentages of switching for dabigatran, rivaroxaban, and apixaban were 5.4, 2.6, and 0.9%, respectively (*P* = 0.014).

**Table 2:** Percentage of potentially inappropriate dosing, switching, and concomitant use of contraindicated drugs of direct oral anticoagulants in patients with atrial fibrillation aged over 65.

	Apixaban, *n* (%)	Dabigatran, *n* (%)	Rivaroxaban, *n* (%)	*P* value
***Overall*, *n***	106	593	535	
***Potentially inappropriate dosing and switching***				
***Dose***^***a***^				
> Standard dose	0 (0)	11 (1.9)	18 (3.4)	0.061
< Recommended low-dose	1 (0.9)	44 (7.4)	72 (13.5)	< 0.001
Total	1 (0.9)	55 (9.3)	90 (16.8)	< 0.001
***Switch***^***b***^	1 (0.9)	32 (5.4)	14 (2.6)	0.014
***Concomitant use of contraindicated drugs***^***c***^				
***LMWH***^***d***^				
Enoxaparin	1 (0.9)	21 (3.5)	57 (10.7)	< 0.001
Dalteparin	0 (0)	1 (0.2)	0 (0)	0.582
***Heparin derivative***^***d***^				
Fondaparinux	0 (0)	0 (0)	0 (0)	-
***OAC***^***d***^				
Warfarin	12 (11.3)	38 (6.4)	33 (6.1)	0.140
Apixaban	-	9 (1.5)	11 (2.1)	0.298
Dabigatran	0 (0)	-	2 (0.4)	0.270
Rivaroxaban	1 (0.9)	25 (4.2)	-	< 0.001
***Potent inhibitor of P-gp***^***e***^* *				
Cyclosporine	-	1 (0.2)	-	-
Itraconazole	-	5 (0.8)	-	-
Dronedarone	-	5 (0.8)	-	-
***Inducer of P-gp***^***e***^* *				
Rifampicin	-	5 (0.8)	-	-
St. John’s wort	-	0 (0)	-	-
Carmazepine	-	6 (1.0)	-	-
Phenytoin	-	3 (0.5)	-	-

LMWH, Low molecular weight heparin; OAC, Oral anticoagulant; P-gp, P-glycoprotein.

^a^Using DOACs at least 8 days at doses which were not recommended in the Summary of Product Characteristics

^b^Using another DOACs rather than the DOAC prescribed at the index date for at least 8 days

^c^Concomitant use of contraindicated drugs for least 1 day were included in this analysis, except for the OAC category (concomitant use of OACs for at least 8 days was included)

^d^Contraindicated drugs with all DOACs

^e^Contraindicated drugs with dabigatran only.

Table 2 shows the percentage of the concomitant use of contraindicated drugs in patients with AF aged over 65 who received DOACs. From a total of 1,234 patients, 236 patients (19.1%) were prescribed contraindicated drugs with an average of 1.89 prescriptions per patient. Among the patients treated with apixaban, 12 patients received concomitant warfarin therapy (11.3%). Among the patients treated with dabigatran, 38 patients (6.4%) were prescribed concomitant warfarin, and 25 patients (4.2%) were prescribed drugs related to P-glycoprotein. Among the rivaroxaban-treated patients, concomitant use of enoxaparin was the most common (10.7%), followed by warfarin (6.1%). In general, concomitant use of other oral anticoagulants was relatively frequent. The frequency of concomitant use of warfarin with DOACs was not significantly different among different DOACs.

In the multivariate logistic model, lower level of medical institution and Chungcheong region were significantly associated with higher odds of concomitant use of contraindicated drugs in patients with AF aged over 65 (Table 3). In addition, outpatient visits were 4.7 times more likely to result in concomitant use of contraindicated drug than inpatient settings.

**Table 3:** Factors associated with concomitant use of contraindicated drugs in patients with atrial fibrillation aged over 65 who received direct oral anticoagulants.

Factors	Odds Ratio (95% CI)^a^
***Level of institution***	
Tertiary hospital	1
General hospital	1.018 (0.691–1.215)
Clinic ∙ Long-term care hospital ∙ Hospital	2.434 (1.215–4.876)*
***Bed size***	
≤ 100	1
101–300	0.382 (0.207–0.704)*
301–800	0.361 (0.178–0.732)*
801–1000	0.232 (0.105–0.516)*
> 1,000	0.298 (0.13–0.684)*
***Region of institution***	
Seoul ∙ Gyeonggi	1
Gangwon	0.723 (0.424–1.232)
Chungcheong	1.563 (1.008–2.423)*
Gyeongsang	1.262 (0.923–1.724)
Jeolla	1.142 (0.832–1.568)
Jeju	0.776 (0.333–1.811)
***Type of hospital visit***	
Inpatient	1
Outpatient	4.718 (3.692–6.028)*

CI, confidence interval.

*Statistically significant, *P* < 0.05

^*a*^*c*-statistic = 0.798.

### Medication adherence

The mean MPR of patients who had at least two DOAC prescriptions was 0.92 (Table 4). The mean MPRs for apixaban, dabigatran, and rivaroxaban were 0.95, 0.93, and 0.91, respectively, which were not significantly different from each other (*P* = 0.075). Non-adherence (MPR < 0.8) was observed in 16.2%, 11.6%, and 8.3% of rivaroxaban, dabigatran, and apixaban users, respectively (*P* = 0.086; Table 4 and S4 Fig). When the mean MPR values were stratified by CHA_2_DS_2_-VASc scores, the mean MPR was the highest in patients with a CHA_2_DS_2_-VASc score of 5.

**Table 4:** Medication adherence to direct oral anticoagulants in patients with atrial fibrillation aged over 65.

	Total	Apixaban	Dabigatran	Rivaroxaban	*P* value*
***Overall*, *n***	921	72	492	357	
***MPR for adherence*, *mean ± SD***					
All	0.92 ± 0.16	0.95 ± 0.12	0.93 ± 0.15	0.91 ± 0.18	0.075
Female	0.92 ± 0.17	0.95 ± 0.14	0.92 ± 0.16	0.91 ± 0.18	0.637
Male	0.92 ± 0.16	0.95 ± 0.11	0.93 ± 0.15	0.90 ± 0.18	0.057
***MPR stratified by CHA***_***2***_***DS***_***2***_***-VASc score*, *mean ± SD***					
< 4	0.90 ± 0.18	0.97 ± 0.11	0.92 ± 0.15	0.86 ± 0.22	0.059
4	0.93 ± 0.15	0.97 ± 0.46	0.93 ± 0.15	0.92 ± 0.16	0.304
5	0.94 ± 0.14	0.91 ± 0.18	0.94 ± 0.12	0.93 ± 0.16	0.526
6	0.91 ± 0.19	0.98 ± 0.05	0.92 ± 0.19	0.89 ± 0.21	0.299
> 6	0.91 ± 0.19	0.91 ± 0.18	0.89 ± 0.21	0.94 ± 0.14	0.695
***Non-adherent patients*, *n (%)***					
	121 (13.1)	6 (8.3)	57 (11.6)	58 (16.2)	0.086

MPR, medication possession ratio; SD, standard deviation.

*Statistically significant, *P* < 0.05.

## Discussion

This study examined the drug utilization patterns (potentially inappropriate dosing, switching, and concomitant use of contraindicated drugs) and medication adherence to DOACs in patients with AF aged over 65 in Korea. To the best of our knowledge, this is the first study to evaluate the real-world use of apixaban, dabigatran, and rivaroxaban in Korea. First, we found that in Korea, the most commonly prescribed DOAC among patients with AF aged over 65 who were unsuitable for warfarin during the whole study period was dabigatran. Second, 16.8% of the patients treated with rivaroxaban received potentially inappropriate dose (higher than the standard doses or lower than the recommended low doses), followed by dabigatran and apixaban (9.3 and 0.9%, respectively). Third, switching was the most frequent in the dabigatran group, whereas it occurred in 2.6 and 0.9% of the patients treated with rivaroxaban and apixaban, respectively. These differences in switching frequency might be attributed to the different launch time of each DOAC or other issues, such as tolerability, which needs further investigation. Fourth, concomitant use of other anticoagulants and enoxaparin was relatively frequent. Lower level of medical institution, Chungcheong region, and outpatient visits were significantly associated with concomitant use of contraindicated drugs. Fifth, medication adherence measured as the mean MPR was relatively high in all DOAC groups, which were not significantly different from each other (0.95, 0.93, and 0.91 for apixaban, dabigatran, and rivaroxaban, respectively).

Dabigatran was the most commonly prescribed DOAC among Korean patients with AF aged over 65 during the whole study period, followed by rivaroxaban and apixaban. This trend was also observed by Olesen *et al*., the Danish nationwide administrative registries study, where dabigatran was the first launched DOAC in Denmark [16]. In Korea, rivaroxaban was launched in July 2009, followed by dabigatran in June 2011, apixaban in April 2013, and edoxaban in February 2016. The market share of DOACs might be owing to the launch time, prescription preference, or characteristics of treatments and included patients. Because this study only included patients with AF unsuitable for warfarin, these inclusion criteria might have affected the prescription trend. In addition, at the time of this study, edoxaban was not available in the dataset that was used in this study. Further investigations with longer follow-up periods are needed to understand the prescription trend and impact of the market entry of edoxaban in 2015.

It should be noted that understanding of the reimbursement criteria is important to interpret the study findings. DOACs were not covered for all AF patients until June 30, 2015 in Korea. They were only reimbursed for patients with AF unsuitable for warfarin due to poor international normalized ratio (INR) control, hypersensitivity or contraindications to warfarin. DOACs started to be reimbursed as the first-line therapy for AF patients from July 1, 2015. Since the available dataset only included claims in 2014, the study findings explain the use of DOACs in patients with AF aged over 65 who were unsuitable for warfarin. Thus, caution is needed when interpreting the study results; in addition, it is not appropriate to extend the study findings to all DOACs users.

Nevertheless, understanding of this special population which is patients with AF unsuitable for warfarin is still important. Because a large proportion of patients with atrial fibrillation (AF) had still received warfarin (44.2% in 2016 in Korea) in the DOAC era, significant number of patients are expected to get warfarin [17]. As time goes by, these warfarin users could become unsuitable for warfarin (e.g., poor INR control, severe kidney/liver disease, severe hypertension). Thus, understanding issues in this population is important and this study provides an insight into the potentially inappropriate anticoagulation therapy in patients with AF unsuitable for warfarin.

For the majority of AF patients included in this study, apixaban, dabigatran, and rivaroxaban were appropriately prescribed (99.1%, 90.7%, and 83.1%, respectively). We also found that using lower dose (< the recommended low dose) of DOACs was more frequent than using higher dose (> the standard dose) of DOACs. Using inappropriate low dose of DOACs is related to an increase in stroke risk [18]. According to the SmPC for apixaban, dabigatran, and rivaroxaban, older age, renal insufficiency, lower body weight, and concomitant use of interacting medications can increase the blood concentration of DOACs, which tends to increase the bleeding risk. Two previous studies reported that these factors might contribute to the use of inappropriate low doses of DOACs [19, 20]. The risks of stroke and bleeding should be balanced out to get the best treatment effects of DOACs. Every drug has both benefits and risks, thus a careful approach is needed to manage each patient. The use of a dose lower than the recommended low dose might increase the risk of stroke, which could endanger the patient. Thus, further research examining the outcomes of using doses lower than the recommended low dose of DOACs is warranted.

Switching to a different DOAC was not prevalent as inappropriate dosing of DOACs. This might be due to the length of the follow-up period, which was less than one year. Zhou *et al*. reported differences in adherence among switchers and non-switchers. Interestingly, they found that the mean MPR values were relatively higher among switchers than among non-switchers [21]. It seemed that switching might be one of the factors associated with adherence to DOACs. However, in this study, we did not compare the adherence between switchers and non-switchers, which might be further investigated.

As mentioned above, the concomitant use of contraindicated drugs can affect the bleeding risk or treatment effectiveness by modifying the blood concentration of DOACs [18]. In our analysis, the concomitant use of enoxaparin and warfarin was relatively frequent. Both drugs have a potential to increase the bleeding risk. A previous study found that approximately 7% of dabigatran users were concomitantly receiving low molecular weight heparin [22]. Thus, a medication monitoring system to detect such contraindicated combinations is warranted to reduce the risk of bleeding.

Our data indicated that the mean MPR values were similar and high for the three DOACs. The differences in the mean MPR among the three DOACs might be attributed to the differences in the dosing interval and adverse reaction profile [23]. However, since we did not find significant differences in the MPR values among the three DOACs, we concluded that the compliance to the three DOACs was high. This might be attributable to their easy administration (oral formulations) and absence of need for careful monitoring in hospital, unlike warfarin. Patients included in this study have already used warfarin, thus they might be aware of the convenience of DOAC administration. Moreover, they might be at high risk since they were unsuitable for warfarin; therefore, they pay more attention to taking their medications. Another study showed that adherent patients had older age and higher CHADS_2_ scores [21]. These results are consistent with our findings.

This study has several limitations. First, the data used in our study, HIRA-APS, did not include laboratory data (e.g., renal function) or body weight data, which did not allow us to evaluate whether the DOAC dose was appropriately reduced or not. Thus, we used the concept of “lower than the recommended low dose”. For example, <15 mg QD rivaroxaban in patients with AF was considered as “lower than the recommended low dose”. Second, we could not determine whether the patients received all medications prescribed to them, which is one of the inherent limitations of studies using claims data. Third, the first DOAC prescription date of the study population could be before January 1, 2014. However, we did not identify the first DOAC prescription date to define OAC-naïve users. Because we thought that if we included only OAC-naïve users, it would be difficult to reflect the results of patients unsuitable for warfarin due to poor INR, who are the major components of the study population. In addition, the follow-up period of this study (the mean follow-up period was 194.44 days) may not long enough to evaluate adherence to DOACs. However, the impact of length of follow-up on MPR may not be significant in our study population aged over 65, because it has been reported that adherence tend to remain consistent over time in older patients with AF (especially patients aged over 70) [24].

## Conclusions

In conclusion, potentially inappropriate dosing of DOACs in terms of dose reduction (lower doses than the recommended low dose) was observed in DOACs users, which cannot be neglected. Medication adherence was generally high among DOACs users. Further research is needed to determine whether this dose reduction led to higher risk of stroke; in addition, studies are required to test whether the high medication adherence lasted for longer follow-up periods and to determine how this might impact the outcomes. Further evaluation of these phenomena in real-world settings will assist clinicians to find the best treatment options for patients who are different from the strictly refined patients included in the clinical trials. Concomitant use of contraindicated drugs was mainly related to the use of other anticoagulants and enoxaparin. This was significantly associated with smaller healthcare institutions and outpatient visits. Therefore, an alert system of concomitant use of contraindicated drugs needs to be implemented.

## Supporting information

S1 Fig Distribution of the number of patients with direct oral anticoagulants stratified by CHA_2_DS_2_-VASc scores. (DOCX)

S2 Fig Distribution of the number of patients with direct oral anticoagulants stratified by The AnTicoagulation and Risk Factors In Atrial Fibrillation (ATRIA) scores. (DOCX)

S3 Fig Monthly number of patients who were prescribed direct oral anticoagulants. (DOCX)

S4 Fig Proportion of adherent patients (medication possession ratio ≥ 0.80). (DOCX)

S1 Table Medication code of direct oral anticoagulants in Korea. (DOCX)

S2 Table ICD-10 codes for CHA_2_DS_2_-VASc score. (DOCX)

S3 Table ICD-10 codes for ATRIA score. (DOCX)

S4 Table ICD-10 codes for charlson comorbidities index. (DOCX)

S5 Table Indications and recommended doses of direct oral anticoagulants in 2014. (DOCX)

S6 Table Diagnosis and procedure codes used to find patients with deep vein thrombosis, pulmonary embolism, and hip or knee replacement. (DOCX)

S7 Table Medication codes of contraindicated drugs of direct oral anticoagulants. (DOCX)
