# Supplementary material for: The treatment pattern and adherence to direct oral anticoagulants in patients with atrial fibrillation aged over 65
Source: PLoS One. 2019 Apr 1;14(4):e0214666. doi: 10.1371/journal.pone.0214666 (PMC6443233; doi:10.1371/journal.pone.0214666)
Supplement: S1 Fig — (DOCX) [file pone.0214666.s001.docx]

**S1 Fig.** Distribution of the number of patients with direct oral anticoagulants stratified by CHA_2_DS_2_-VASc scores.
