# Supplementary material for: The treatment pattern and adherence to direct oral anticoagulants in patients with atrial fibrillation aged over 65
Source: PLoS One. 2019 Apr 1;14(4):e0214666. doi: 10.1371/journal.pone.0214666 (PMC6443233; doi:10.1371/journal.pone.0214666)
Supplement: S1 Table — (DOCX) [file pone.0214666.s005.docx]

**S1 Table.** Medication code of direct oral anticoagulants in Korea.

| **INN** | **Medication code** |
| --- | --- |
| Apixaban | 617001ATB |
|  | 617002ATB |
| Dabigatran etexilate | 613701ACH |
|  | 613702ACH |
| Rivaroxaban | 511401ATB |
|  | 511402ATB |
|  | 511403ATB |
|  | 511404ATB |

INN, International Nonproprietary Name.
