# Supplementary material for: The treatment pattern and adherence to direct oral anticoagulants in patients with atrial fibrillation aged over 65
Source: PLoS One. 2019 Apr 1;14(4):e0214666. doi: 10.1371/journal.pone.0214666 (PMC6443233; doi:10.1371/journal.pone.0214666)
Supplement: S2 Table — (DOCX) [file pone.0214666.s006.docx]

**S2 Table.** ICD-10 codes for CHA_2_DS_2_-VASc score.

|  | **Risk factors** | **Score** | **ICD-10** |
| --- | --- | --- | --- |
| C | Congestive heart failure/LV dysfunction | 1 | I50 |
| H | Hypertension | 1 | I10–I15 |
| A_2_ | Age≥75 | 2 |  |
| D | Diabetes mellitus | 1 | E10–14 |
| S_2_ | Stroke/TIA/TE | 2 | I69, I74, I63, I64, G45, I26 |
| V | Vascular disease (prior MI, PAD, aortic plaque) | 1 | I21, I252, I70–73 |
| A | Age 65-74 | 1 |  |
| S | Sex, Female | 1 |  |

ICD-10, *International Classification of Diseases, Tenth Revision*; LV, left ventricular; TIA, transient ischemic attack; TE, thromboembolism; MI, myocardial infarction; PAD, peripheral artery disease.
