# Supplementary material for: The treatment pattern and adherence to direct oral anticoagulants in patients with atrial fibrillation aged over 65
Source: PLoS One. 2019 Apr 1;14(4):e0214666. doi: 10.1371/journal.pone.0214666 (PMC6443233; doi:10.1371/journal.pone.0214666)
Supplement: S3 Table — (DOCX) [file pone.0214666.s007.docx]

**S3 Table.** ICD-10 codes for ATRIA score.

| **Risk factor** | **Score** | **ICD-10** |
| --- | --- | --- |
| Anemia | 3 | D46, D50-53, D55-61, D63-64 |
| Severe renal disease | 3 | I12.0, I13.1, I13.2, N03-05, N18-19, P96.0, T82.4, Y84.1, Z99.2 |
| Age≥75 | 2 |  |
| Prior Bleeding | 1 | I60-62, K25.0, K25.2, K25.4, K25.6, K92.2 |
| Hypertension | 1 | I10–13 |

ICD-10, *International Classification of Diseases, Tenth Revision*; ATRIA, The AnTicoagulation and Risk Factors In Atrial Fibrillation.
