# Supplementary material for: The treatment pattern and adherence to direct oral anticoagulants in patients with atrial fibrillation aged over 65
Source: PLoS One. 2019 Apr 1;14(4):e0214666. doi: 10.1371/journal.pone.0214666 (PMC6443233; doi:10.1371/journal.pone.0214666)
Supplement: S4 Table — (DOCX) [file pone.0214666.s008.docx]

**S4 Table.** ICD-10 codes for Charlson Comorbidities Index.

| **Comorbidities** | **Weight** | **ICD-10** |
| --- | --- | --- |
| Cerebrovascular disease | 1 | G45.x, G46.x, H34.0, I60.x-I69.x |
| Congestive heart failure | 1 | I09.9, I11.0, I13.0, I13.2, I25.5, I42.0, I42.5-I42.9, I43.x, I50.x, P29.0 |
| Chronic pulmonary disease | 1 | I27.8, I27.9, J40.x–J47.x, J60.x–J67.x, J68.4, J70.1, J70.3 |
| Dementia | 1 | F00.x–F03.x, F05.1, G30.x, G31.1 |
| Diabetes without chronic complication | 1 | E10.0, E10.1, E10.6, E10.8, E10.9, E11.0, E11.1, E11.6, E11.8, E11.9, E12.0, E12.1, E12.6, E12.8, E12.9, E13.0, E13.1, E13.6, E13.8, E13.9, E14.0, E14.1, E14.6, E14.8, E14.9 |
| Mild liver disease | 1 | B18.x, K70.0-K70.3, K70.9, K71.3-K71.5, K71.7, K73.x, K74.x, K76.0, K76.2-K76.4, K76.8, K76.9, Z94.4 |
| Myocardial infarction | 1 | I21.x, I22.x, I25.2 |
| Peripheral vascular disease | 1 | I70.x, I71.x, I73.1, I73.8, I73.9, I77.1, I79.0, I79.2, K55.1, K55.8, K55.9, Z95.8, Z95.9 |
| Peptic ulcer disease | 1 | K25.x–K28.x, |
| Rheumatologic disease | 1 | M05.x, M06.x, M32.x–M34.x M31.5M35.1, M35.3, M36.0 |
| Diabetes with chronic complication | 2 | E10.2–E10.5, E10.7, E11.2–E11.5, E11.7, E12.2-E12.5, E12.7, E13.2–E13.5, E13.7, E14.2–E14.5, E14.7 |
| Hemiplegia or paraplegia | 2 | G04.1, G11.4, G80.1, G80.2, G81.x, G82.x, G83.0-G83.4, G83.9 |
| Any malignancy, including leukemia and lymphoma | 2 | C00.x–C26.x, C30.x–C34.x, C37.x–C41.x, C43.x, C45.x–C58.x, C60.x–C76.x, C81.x–C85.x, C88.x, C90.x–C97.x, |
| Renal disease | 2 | I12.0, I13.1, N03.2-N03.7, N05.2-N05.7, N18.x, N19.x, N25.0, Z49.0-Z49.2, Z94.0, Z99.2 |
| Moderate or severe liver disease | 3 | I85.0, I85.9, I86.4, I98.2, K70.4, K71.1, K72.1, K72.9, K76.5, K76.6, K76.7 |
| AIDS/HIV | 6 | B20.x–B22.x, B24.x |
| Metastatic solid tumor | 6 | C77.x–C80.x |

ICD-10, *International Classification of Diseases, Tenth Revision*.
