# Supplementary material for: The treatment pattern and adherence to direct oral anticoagulants in patients with atrial fibrillation aged over 65
Source: PLoS One. 2019 Apr 1;14(4):e0214666. doi: 10.1371/journal.pone.0214666 (PMC6443233; doi:10.1371/journal.pone.0214666)
Supplement: S5 Table — (DOCX) [file pone.0214666.s009.docx]

**S5 Table.** Indications and recommended doses of direct oral anticoagulants in 2014.

| **Indications** | **Recommended doses for each indication [1-4]** | | |
| --- | --- | --- | --- |
|  | **Apixaban** | **Dabigatran** | **Rivaroxaban** |
| To decrease risk of stroke and systemic embolism in patients with nonvalvular atrial fibrillation | 5 mg or 2.5 mg^a^ twice daily | 150 mg or 110 mg^b^ twice daily | 20 mg or 15 mg^c^ once daily |
| To prevent venous thromboembolism after total hip or knee replacement surgery | 2.5 mg twice daily |  | 10 mg once daily (5 weeks for hip replacement and 2 weeks for knee replacement) |
| To treat and prevent deep venous thrombosis and pulmonary embolism |  |  | 15 mg twice daily (the first 3 weeks after occurrence) or 20 mg once daily (for maintenance or preventing recurrence) |
| ^a^Apixaban 2.5 mg twice daily should be considered for patients who have renal dysfunction (creatinine clearance (CrCl), 30-49 mL/min) or who have two or more of the following three factors: elderly patients (≥ 80 years old), body weight (≤ 60 kg), or serum creatinine level (≥ 1.5 mg/dL); ^b^Dabigatran 110 mg twice daily should be considered in the following cases: elderly patients (≥ 80 years old), moderate renal dysfunction (CrCl, 30-49 mL/min), or high bleeding risk (HAS-BLED score ≥ 3), or concomitant administration of interacting drugs (e.g., verapamil); ^c^Rivaroxaban 15 mg once daily should be considered in the following cases: moderate renal dysfunction (CrCl, 30-49 mL/min), or high bleeding risk (HAS-BLED score ≥ 3). | | | |

**References**

1. Summary of Product Characteristics (INN – rivaroxaban). Available at: https://www.ema.europa.eu/documents/product-information/xarelto-epar-product-information_en.pdf. Accessed 2 Jan 2019.

2. Summary of Product Characteristics (INN – dabigatran etexilate). Available at: https://www.ema.europa.eu/documents/product-information/pradaxa-epar-product-information_en.pdf. Accessed 2 Jan 2019.

3. Summary of Product Characteristics (INN – apixaban). Available at: https://www.ema.europa.eu/documents/product-information/eliquis-epar-product-information_en.pdf. Accessed 2 Jan 2019.

4. Jung BC, Kim NH, Nam GB, Park HW, On YK, Lee YS, et al. The Korean Heart Rhythm Society's 2014 statement on antithrombotic therapy for patients with nonvalvular atrial fibrillation: Korean Heart Rhythm Society. Korean Circ J. 2015;45(1):9-19.
