# Supplementary material for: The treatment pattern and adherence to direct oral anticoagulants in patients with atrial fibrillation aged over 65
Source: PLoS One. 2019 Apr 1;14(4):e0214666. doi: 10.1371/journal.pone.0214666 (PMC6443233; doi:10.1371/journal.pone.0214666)
Supplement: S6 Table — (DOCX) [file pone.0214666.s010.docx]

**S6 Table.** Diagnosis and procedure codes used to find patients with deep vein thrombosis, pulmonary embolism, and hip or knee replacement.

| **Disease/surgery** | **Codes** | **Sources** |
| --- | --- | --- |
| Deep venous thrombosis and pulmonary embolism | I80.2, I80.3, I26.x | ICD-10 codes |
| Hip or knee replacement surgery | N0711, N1711, N1721, N2072, N3712, N3722 | Korean procedure codes |

ICD-10, *International Classification of Diseases, Tenth Revision*.
