# Supplementary material for: The treatment pattern and adherence to direct oral anticoagulants in patients with atrial fibrillation aged over 65
Source: PLoS One. 2019 Apr 1;14(4):e0214666. doi: 10.1371/journal.pone.0214666 (PMC6443233; doi:10.1371/journal.pone.0214666)
Supplement: S7 Table — (DOCX) [file pone.0214666.s011.docx]

**S7 Table.** Medication codes of contraindicated drugs of direct oral anticoagulants.

| **Categories** | **Medication** | **Medication codes** |
| --- | --- | --- |
| Contraindication to apixaban, dabigatran, and rivaroxaban | Enoxaparin | 152101BIJ, 152102BIJ, 152103BIJ, 152105BIJ, 152104BIJ, 152106BIJ |
|  | Dalteparin | 140201BIJ, 140202BIJ, 140203BIJ |
|  | Fondaparinux | 450101BIJ |
|  | Warfarin | 249103ATB, 249105ATB |
|  | Apixaban | 617002ATB, 617001ATB |
|  | Dabigatran | 613702ACH, 613701ACH |
|  | Rivaroxaban | 511403ATB, 511402ATB, 511401ATB, 511404ATB |
| Contraindication to dabigatran | ***Potent inhibitor of P-gp*** | |
|  | Cyclosporine | 194701ALQ, 194701ACS, 194702ACS, 139202BIJ, 139201ACS, 139204ACS |
|  | Itraconazole | 179101ATB, 179104ATB, 179101ACH, 179101ATB, 179102ALQ, 179103BIJ |
|  | Dronedarone | 597401ATB |
|  | ***Inducer of P-gp*** | |
|  | Rifampicin | 223904ATB, 223901ACH, 223902ACH, 223902ATB, 223903ATB, 489200ATB, 519500ATB, 380200ATB |
|  | St. John’s wort | 149901ATB |
|  | Carbamazepine | 123102ATB, 123102ATR, 123104ATR |
|  | Phenytoin | 146801ATB, 146801ACH, 146801BIJ, 146802BIJ |
| P-gp, P-glycoprotein. | | |
